# A Novel Strategy Involved Anti-Oxidative Defense: The Conversion of NADH into NADPH by a Metabolic Network

**DOI:** 10.1371/journal.pone.0002682

**Published:** 2008-07-16

**Authors:** Ranji Singh, Joseph Lemire, Ryan J. Mailloux, Vasu D. Appanna

**Affiliations:** Department of Chemistry and Biochemistry, Laurentian University, Sudbury, Ontario, Canada; University of Arkansas for Medical Sciences, United States of America

## Abstract

The reduced nicotinamide adenine dinucleotide phosphate (NADPH) is pivotal to the cellular anti-oxidative defence strategies in most organisms. Although its production mediated by different enzyme systems has been relatively well-studied, metabolic networks dedicated to the biogenesis of NADPH have not been fully characterized. In this report, a metabolic pathway that promotes the conversion of reduced nicotinamide adenine dinucleotide (NADH), a pro-oxidant into NADPH has been uncovered in *Pseudomonas fluorescens* exposed to oxidative stress. Enzymes such as pyruvate carboxylase (PC), malic enzyme (ME), malate dehydrogenase (MDH), malate synthase (MS), and isocitrate lyase (ICL) that are involved in disparate metabolic modules, converged to create a metabolic network aimed at the transformation of NADH into NADPH. The downregulation of phosphoenol carboxykinase (PEPCK) and the upregulation of pyruvate kinase (PK) ensured that this metabolic cycle fixed NADH into NADPH to combat the oxidative stress triggered by the menadione insult. This is the first demonstration of a metabolic network invoked to generate NADPH from NADH, a process that may be very effective in combating oxidative stress as the increase of an anti-oxidant is coupled to the decrease of a pro-oxidant.

## Introduction

NADPH is an essential anabolic reducing agent in all living organisms and it is involved in a plethora of biochemical reactions. NADPH is vital in anti-oxidative defense mechanisms as it is the universal reducing power fuelling the activities of such enzymes as catalase, superoxide dismutase, and glutathione peroxidase [1]. These proteins play a crucial role in allowing organisms to thrive in an aerobic environment. The biogenesis of DNA is another important function mediated by NADPH in all organisms. Without the involvement of this cofactor, ribonucleotide reductase will be unable to convert ribose nucleotides into their deoxy counterparts. The biosynthesis of lipids also necessitates the participation of this moiety. Only recently, the role of this pyridine dinucleotide in signaling processes has begun to emerge [2], [3]. Owing to its involvement in a multitude of functions, NADPH is undoubtedly a very critical molecule in all living organisms.

Hence, it is not surprising that living systems have evolved numerous intricate strategies to acquire this important pyridine dinucleotide. Isocitrate dehydrogenase (ICDH)-NADP^+^ dependent, glucose-6-phosphate dehydrogenase (G6PDH), 6-phosphogluconate dehydrogenase (6PGDH), ME and glutamate dehydrogenase-NADP^+^ dependent (GDH) are the main enzymes earmarked for the genesis of NADPH [4], [5]. Although the significance of G6PDH in oxidative stress has been known, only recently has the importance of ICDH in the detoxification of reactive oxygen species (ROS) been delineated. In the mitochondria and the peroxisomes, this enzyme appears to be the main generator of NADPH [6]–[8]. Alternative routes for the production of NADPH are also operative in a variety of organisms. Pyridine nucleotide transhydrogenase enables the formation of NADPH from NADH. This is powered by the proton motive force and involves the transfer of a hydride from NADH to NADP with the concurrent production of NADPH [9]. The direct phosphorylation of NADH to NADPH, a process necessitating the utilization of ATP and mediated by the NADH kinase has also been shown to contribute to the homeostasis of the reducing agent [2]. Even though these processes are important in satisfying the cellular needs in NADPH, it has been argued that these enzymes will be ineffective if the supply of the precursor NADP is inadequate. Recently, we have demonstrated how the enzyme NAD kinase is central in combating oxidative stress. This enzyme converts NAD to NADP and helps orchestrate the production of NADPH, a powerful reductive force at the expense of NADH, a pro-oxidant [10].

Although these one-step enzymatic processes involved in the biogenesis of NADPH have been extensively studied, the participation of metabolic networks dedicated to the maintenance of the intracellular concentration of NADPH has not been fully delineated. Due to the central role of this moiety in anti-oxidative defense mechanisms, such a metabolic module would provide enormous reductive flexibility to the cells. As part of our study to elucidate the various stratagems organisms deploy to live in an aerobic environment and to survive oxidative stress, we have identified an intriguing metabolic pathway dedicated to the production of NADPH. Here, we show that metabolic modules normally associated with gluconeogenesis, glycolysis, the tricarboxylic acid cycle and the glyoxylate cycle coalesce to create a unique network that converts NADH to NADPH. The significance of MDH, ME, PC, and PEPCK in the formation of this unique metabolic module is also discussed.

## Materials and Methods

### Microbial growth conditions and cellular fractionation

*Pseudomonas fluorescens* (ATCC 13525) were grown in a mineral medium containing citrate as the sole carbon source as described previously [10]. Prior to inoculation, the media was dispensed in 200 mL aliquots and autoclaved. Menadione (100 µM) was added to the medium following sterilization. Cells from citrate (control) and menadione-containing cultures were isolated at similar growth phases for analysis (25 h for control and 30 h for menadione-stress). Bacterial cells were harvested as described previously and suspended in a cell storage buffer (CSB; 50 mM Tris-HCl, 5 mM MgCl_2_, 1 mM phenylmethylsulfonylfluoride, pH 7.3) [11]. Cells were disrupted by sonication and subjected to centrifugation at 3000×g to remove any intact cells. The cell-free extract (CFE) was then centrifuged for 3 h at 180, 000×g to afford a soluble CFE fraction and a membranous CFE fraction. Purity of the fractions was verified by monitoring G6PDH and complex I activity in the membrane and soluble CFE. The protein content of both fractions was determined using the Bradford assay [12]. BSA served as the protein standard.

### Regulation experiments

Regulation experiments were performed as described previously [10]. 10 mg of protein equivalent of menadione-stressed cells were transferred to citrate (control) media, and 10 mg of protein equivalent of control cells were transferred to menadione-supplemented (100 µM) media. Following an incubation for 8 h, cells were harvested and the cellular fractions were isolated and assayed for enzymatic activities. To afford a proper comparison, control cells grown for 25 h and menadione cells grown for 30 h were used in the regulation experiment.

### Blue Native (BN)-PAGE, in-gel activity assays, and two-dimensional (2D) BN-PAGE

Blue Native polyacrylamide gel electrophoresis (BN PAGE) and in-gel activity stains were performed as described previously [11], [13]. Membrane proteins were prepared in a Native buffer (50 mM BisTris, 500 mM ε-aminocaproic acid (pH 7.0)) containing 1% maltoside at a final concentration of 4 mg/mL. Soluble proteins were prepared in a similar manner except maltoside was omitted. MDH and ME were assayed as described previously using malate and NAD(P) as substrates. The activities of PC and PEPCK were tested using an enzyme-coupled assay as described in [14]. 16.7 µg/mL of 2,4-dichloroindophenol (DCIP) replaced PMS as the electron mediator for these assays. The in-gel activity of MS was also tested using an enzyme-coupled assay. The activity of MS was visualized using reaction buffer, 5 mM glyoxylate, 5 mM acetyl-CoA, 0.5 mM NAD, 5units of MDH, PMS, and INT. The activity of ICL was also assayed by coupling the formation of glyoxylate to exogenously added LDH type II [15]. The activity band for ICL was made apparent using reaction buffer, 5 mM isocitrate, 0.5 mM NAD, 10units of LDH, INT, and PMS. The in-gel activities of PK and PDH were detected as described previously [10]. The activity was stopped using destaining solution (40% methanol, 10% acetic acid) once the bands reached their desired intensity. The specificity of the activity bands was confirmed by running known standards and by performing the reactions in the absence of substrates. Proper loading was assured by Coomassie staining for total protein. BSA was used as a molecular mass (MM) marker. Activity bands were quantified using SCION Imagine for Windows (SCION Imaging Corp.). For 2D BN-PAGE, activity bands were precision cut and loaded vertically into a 4–16% linear gradient gel. Electrophoresis was carried out as described above. Proteins were detected by Coomassie staining.

### Two-dimensional (2D) electrophoresis and immunoblotting

SDS-PAGE and 2D SDS-PAGE gels were performed in accordance with the modified method described in [14], [16]. For SDS-PAGE and immunoblot, 30 µg of protein was solubilized in 62.5 mM Tris-HCl (pH 6.8), 2% SDS, and 2% β-mercaptoethanol at 100°C for 5 min. Following solubilization the protein samples were electrophoresed in a 10% isocratic denaturing gel using a discontinuous buffer system. The proteins were then electroblotted to a Hybond™-Polyvinylidene difluoride membrane for immunoblotting as described in [11]. Avidin antibodies (Sigma) directed against biotin was used to ascertain the levels of PC. Kaleidoscope molecular mass markers (Bio-Rad) were used to estimate the molecular mass. Following several washings the membrane was dried and probed for 5 min at room temperature with Chemiglow reagent (Alpha Innotech). The immunoblot was documented using a ChemiDoc XRS system (Biorad Imaging Systems). For 2D SDS-PAGE analysis, activity bands from native gels were precision cut and incubated in denaturing buffer (1% β-mercaptoethanol, 5% SDS) for 30 min, and then loaded vertically into the SDS gel. Electrophoresis was carried out as described above. Proteins were detected using a Silver staining kit (Bio-Rad).

### Metabolite analysis

The levels of NADH, NADPH, oxaloacetate, malate, and pyruvate were ascertained by HPLC. Soluble CFE from control and menadione-treated cells were diluted to 2 mg of protein equivalent/mL in ddH_2_O and then boiled for 2 min. Following the removal of the precipitate, the supernatant was injected into an Alliance HPLC equipped with a C_18_-reverse phase column (Synergi Hydro-RP; 4 µm; 250×4.6 mm, Phenomenex) operating at a flow rate of 0.7 mL/min for organic acids and 0.2 mL/min for nicotinamide dinucleotides [17]. Metabolites were detected using a dual wavelength absorbance detector operating at 210 nm for organic acids and 254 nm for nucleotides. 20 mM KH_2_PO_4_ (pH 2.9) was the mobile phase for organic acids while a 95% 20 mM KH_2_PO_4_: 5% acetonitrile (pH 7) was favoured as the mobile phase for the reduced nicotinamide dinucleotides. Metabolites were identified by injecting known standards and the peaks were quantified using EMPOWER software (Waters Corp.). The HPLC was standardized using a 5-point calibration prior to each injection.

The cycling of pyruvate for the conversion of NADH into NADPH was monitored by performing two separate reactions. The metabolism of oxaloacetate to pyruvate by MDH and ME was monitored by reacting 0.2 mg of protein equivalent to soluble CFE with 10 mM oxaloacetate, 1 mM ATP, and 1 mM NADH for 60 min. A similar reaction was performed with membrane fraction to assess the conversion of pyruvate back to oxaloacetate by PC. Reactions were performed by incubating 0.2 mg/mL of protein equivalent to membrane CFE in a reaction buffer containing 10 mM pyruvate, 1 mM GTP, and 1 mM HCO_3_^−^ for 60 min. Reactions were quenched at different time intervals by boiling and then injected into the HPLC. Oxaloacetate, pyruvate, NADH, and NADPH were identified as described above. Negative reactions were performed in the absence of either ATP or GTP. The conversion of NADH into NADPH by MDH and ME was confirmed using 1 mM 3-bromopyruvate, a known inhibitor of ME [18].

### Statistical analysis

Data were expressed as mean±SD. Statistical correlations of data were checked for significance using the student t test. Experiments were performed twice and in triplicate.

## Results

### Metabolite analysis

**Figure 1:**
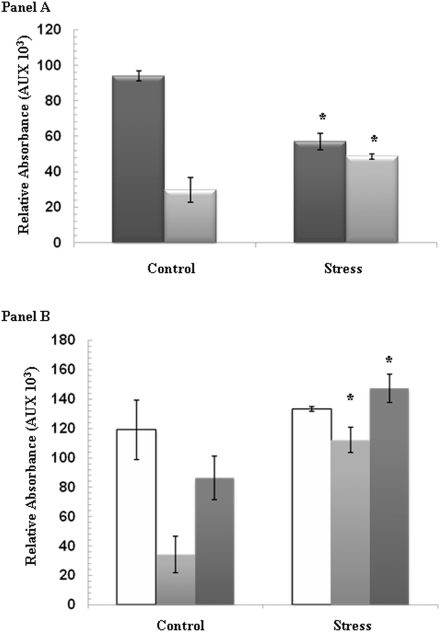
Metabolite profile of *P.fluorescens* following menadione insult. HPLC analysis was performed on the CFE from *P.fluorescens* exposed to control or menadione-stressed conditions. Panel A) Analysis of NADH and NADPH levels. Dark gray bars = NADH, Light gray bars = NADPH. Panel B) Analysis of pyruvate, oxaloacetate, and malate levels. White bars = oxaloacetate, Light gray bars = pyruvate, and Dark gray bars = malate. n = 3, p≤0.05, mean±S.D. * represents statistical significance relative to the control.

*P.fluorescens* exposed to menadione displayed a sharp increase in NADPH levels and a concomitant decrease in NADH (Figure 1, Panel A). This data is consistent with our previous observations that *P. fluorescens* treated with menadione manipulates the levels of NADH and NADPH in an effort combat oxidative stress [10]. HPLC analyses of pyruvate, oxaloacetate, and malate levels provided further insight into the metabolic shift observed following menadione treatment. In contrast to the control cells, the menadione-treated cells accumulated malate and pyruvate, two key TCA cycle intermediates (Figure 1, Panel B). Oxaloacetate levels did not appear to vary significantly between the control and menadione-exposed cells. Thus, the drastic alterations in the amounts of both reduced pyridine nucleotides and the accumulation of malate and pyruvate prompted us to investigate the metabolic changes that would induce such as shift.

### Malate-metabolizing enzymes

**Figure 2:**
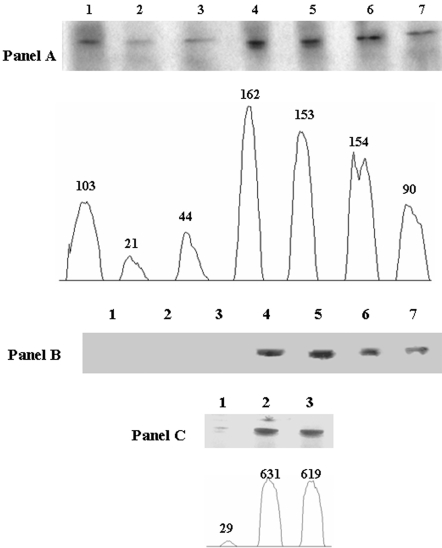
Panel A) In-gel detection of MDH in *P. fluorescens* grown in control and menadione- stress conditions. *Lanes 1, 2, and 3* correspond to the membrane CFE from cells grown in control media for 15, 24, and 30 hrs. *Lanes 4, 5, 6, and 7* correpond to the membrane CFE from cells grown in a menadione-stressed media for 25, 30, 35, and 40 hrs. Bands were quantified using SCION Imaging Software. Panel B) In-gel detection of ME activity. *Lanes 1, 2, and 3* correspond to soluble CFE from the cells grown in control medium for 25, 30, and 35 hrs. *Lanes 4, 5, 6, and 7* correspond to soluble CFE from cells grown in menadione-stressed medium for 30, 35, 40, and 45 hrs. Panel C) Regulation of ME activity. *Lanes 1, 2, and 3* correspond to the soluble control CFE, soluble menadione-stress CFE, and soluble CFE from control cells transferred into a menadione-stress media. Bands were quantified using SCION Imaging Software. Cells were isolated at similar growth phases (25 h for control and 30 h for menadione) unless otherwise indicated.

To decipher the impact of menadione on malate metabolism, we tested the activities and expression of several enzymes involved in the homeostasis of this TCA cycle intermediate. In contrast to control, the menadione-treated cells displayed an increase in MDH activity (Figure 2, Panel A). Indeed, sharp activity bands were recorded in menadione-exposed cells isolated from 25 h to 40 h, respectively. In contrast, the activity bands generated by the control cells isolated at different time intervals were not as intense. Similar to MDH, ME also displayed a drastic increase in activity in menadione-exposed cells (Figure 2, Panel B). Indeed, intense activity bands were documented in *P. fluorescens* exposed to menadione for 25 h to 40 h. Control cells displayed no discernable ME activity. This metabolic shift would account for the high levels of pyruvate and NADPH in the menadione-treated cells. Regulation experiments provided further insight into the ability of menadione to enhance ME activity. Exposure of the control cells to a menadione-containing medium for 8 h led to a sharp increase in ME activity (Figure 2, Panel C).

### The modulation of PC, PEPCK, PK, and PDH

**Figure 3:**
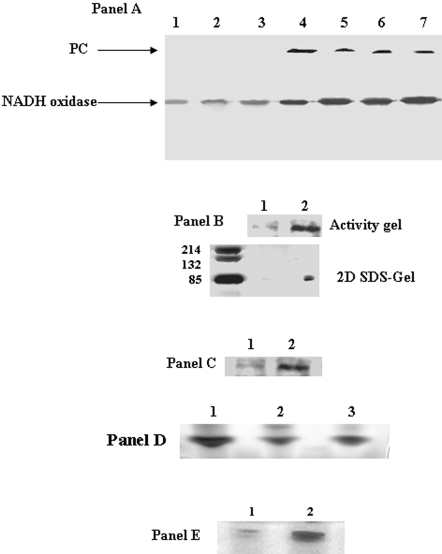
Determination of PC, PEPCK, and PK activity and expression in control and menadione-stressed cells. Panel A) In-gel detection of PC activity. *Lanes 1, 2, and 3* correspond to membrane CFE from cells grown in control media for 15, 24, and 30 hrs. *Lanes 4, 5, 6, and 7* correspond to membrane CFE from cells grown in menadione-stressed media for 25, 30, 35, and 40 hrs. Panel B) 2D SDS-PAGE analysis of PC expression. In-gel activity bands were excised and resolved by 2D SDS-PAGE to determine protein levels. *Lane 1 and 2* correspond to control and menadione-stress cultures. Proteins were detected by silver staining. Panel C) Immunoblot analysis of PC expression. *Lane 1 and 2* correspond to the control and menadione-stress cultures. Panel D) Regulation of PEPCK activity. *Lane 1, 2, and 3* correspond to soluble control CFE, soluble menadione-stress CFE, and soluble CFE of control cells transferred into a menadione-stress media. Panel E) In-gel activity analysis of PK. *Lane 1*: corresponds to control and *Lane 2*: corresponds to menadione-stressed soluble CFE. Cells were isolated at similar growth phases (25 h for control and 30 h for menadione) unless otherwise indicated.

The accumulation of pyruvate and the high levels of oxaloacetate prompted us to assess the impact of menadione on two key gluconeogenic enzymes, PC and PEPCK. Cells exposed to menadione for 25 h displayed a sharp increase in PC activity (Figure 3, Panel A). Furthermore, the activity of PC remained elevated following exposure to menadione for up to 40 h. In an effort to determine the amount of protein associated with the activity bands, the activity bands were excised and subjected to 2D SDS-PAGE. Silver staining revealed high amounts of protein associated with the activity bands from the menadione-treated cells (Figure 3, Panel B). The enhanced expression of PC was confirmed by immunoblot. Indeed, avidin-HRP antibodies disclosed high amounts of PC in *P. fluorescens* exposed to menadione (Figure 3, Panel C). In contrast to PC, the activity of PEPCK was decreased in the menadione-treated cells (Figure 3, Panel D). The decreased in PEPCK activity appeared to be dependent on the presence of menadione since this enzyme was diminished in control cells exposed to menadione for 8 h (Figure 3, Panel D). We also tested the activity of other pyruvate-metabolizing enzymes. PK activity was higher in the menadione-treated cells in contrast to control (Figure 3, Panel E). In contrast, PDH displayed a decrease in activity in the menadione-exposed cells [10].

### Oxaloacetate cycling and the conversion of NADH into NADPH

**Figure 4:**
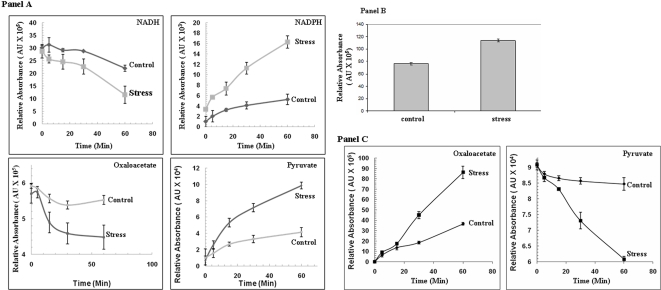
HPLC analysis of the conversion of NADH into NADPH. Cells exposed to control and menadione-stressed conditions were isolated and the soluble and membrane fractions were utilized to assess the conversion of NADH into NADPH. Panel A) The soluble CFE from *P. fluorescens* grown in control and menadione-stressed media were was incubated for 60 min in a reaction mixture containing 10 mM oxaloacetate, 1 mM ATP, and 1 mM NADH. The levels of NADH, NADPH, oxaloacetate, and pyruvate were monitored at various time points by HPLC. Peaks were quantified using EMPOWER software. n = 3, p≤0.05, mean±S.D. Panel B) ME inhibition promotes malate accumulation in menadione-treated cells. The soluble CFE from *P. fluorescens* grown in control and menadione-stressed conditions were incubated in a reaction mixture containing 10 mM oxaloacetate, 1 mM ATP, 1 mM NADH, and 1 mM 3-bromopyruvate. Following a 60 min incubation, the levels of malate were quantified using EMPOWER software. n = 3, p≤0.05, mean±S.D. Panel C) The membrane CFE from control and menadione-stressed cells was incubated for 60 min in a reaction buffer containing 1 mM GTP, and 1 mM HCO_3_^−^. The levels of pyruvate and oxaloacetate were monitored at various time points by HPLC. Oxaloacetate and pyruvate were identified by injecting known standards. Peaks were quantified using EMPOWER software. n = 3, p≤0.05, mean±S.D. Cells were were isolated at 25 h for control and 30 h for menadione-stressed conditions to afford a proper comparison.

The aforementioned data suggest that *P. fluorescens* exposed to menadione alter several key metabolic pathways in order to produce a novel metabolic cycle aimed at converting NADH into NADPH. ME converts malate into pyruvate generating NADPH. Pyruvate is then subsequently converted back into malate with the aid of PC and MDH, a process which requires the oxidation of NADH. The decrease in PEPCK ensures that oxaloacetate is funneled towards malate production. In order to account for the formation of this novel metabolic module, HPLC experiments were performed with the soluble CFE and membrane CFE from the control and menadione-treated cells in two separate stages. In contrast to control, the soluble CFE from the menadione-treated cells readily metabolized oxaloacetate to pyruvate (Figure 4, Panel A). The enhanced conversion of oxaloacetate to pyruvate was mirrored by sharp changes in NADH and NADPH levels. Indeed, the soluble CFE from the menadione-exposed cells contained lower amounts of NADH and higher levels of NADPH following a 60 min incubation in 10 mM oxaloacetate (Figure 4, Panel A). Reactions performed with 3-bromopyruvate also pointed towards the formation of this unique metabolic module. Indeed, treatment with this ME inhibitor resulted in the accumulation of malate in the menadione-treated cells (Figure 4, Panel B). Similar results were also obtained with reactions performed in the absence of ATP; thus pointing to a pivotal role for NADK (data not shown). Hence, MDH and ME work in tandem to convert NADH into NADPH. When the membrane CFE was incubated with pyruvate, GTP, and HCO_3_^−^, the membrane CFE from the menadione-stressed cells converted most of the pyruvate into oxaloacetate (Figure 4, Panel C). In contrast, control membrane CFE converted significantly less pyruvate into oxaloacetate. Reactions performed in the absence of GTP confirmed the observed increase in pyruvate metabolism in the menadione-exposed membrane CFE (data not shown).

### Contribution of ICL and MS to malate production

**Figure 5:**
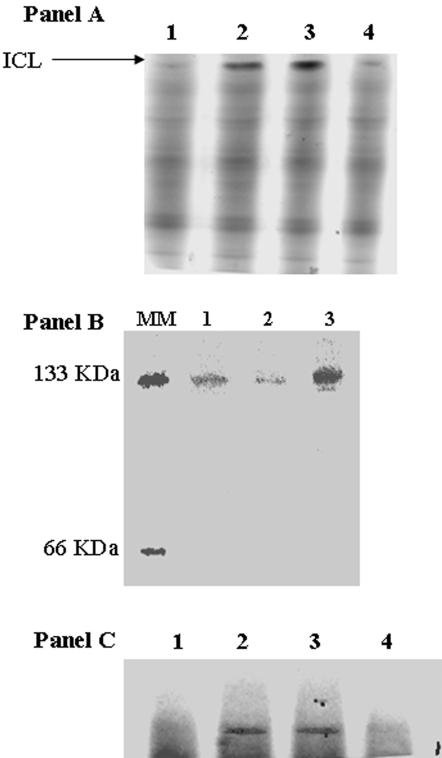
In-gel detection of ICL and MS activity. Panel A: The influence of menadione on ICL activity: *Lanes 1, 2, 3, and 4* correspond to soluble CFE from cells grown in a control medium, control cells transferred into a menadione medium, cells grown in a menadione medium, and menadione-stressed cells transferred to a control medium. Panel B: Assessment of ICL protein levels. Activity bands were excised and resolved on a 2D-Native PAGE. *Lanes 1, 2, and 3* correspond to ICL purchased from Sigma, soluble CFE from control cells, and soluble CFE from menadione-stressed cells. Panel C: In-gel detection of MS activity. *Lanes 1, 2, 3, and 4* correspond to the soluble CFE from cells grown in control media, control cells transferred into menadione-stressed media, menadione media, and menadione-stressed cells transferred into control media. Cells were isolated at similar growth phases (25 h for control and 30 h for menadione) unless otherwise indicated.

Since citrate was the sole carbon source in the bacterial growth medium, we hypothesized that the glyoxylate shunt played an important part in converting citrate to malate. BN-PAGE analysis revealed that ICL activity was enhanced in the menadione-exposed cells. Indeed, a more intense activity band was generated by the menadione-treated cells (Figure 5, Panel A). The alterations in ICL activity also appeared to be dependent on the presence of menadione. Exposure of control cells to a menadione-containing medium for 8 h led to an increase in ICL activity while the opposite trend was observed for menadione-treated cells exposed to a control medium (Figure 5, Panel A). 2D BN-PAGE and Coomassie staining indicated that the enhanced ICL activity in the menadione-treated cells was attributed to an increased amount of protein. Indeed, the activity bands from the menadione-exposed cells contained higher amounts of protein compared to their control counterparts (Figure 5, Panel B). The detection of ICL in aluminum-treated cells served as a positive control [11]. The activity of ICL was complemented by an increase in MS activity. Menadione-treated cells produced more intense activity bands corresponding to MS than the control cells (Figure 5, Panel C). Furthermore, the exposure of control cells to a menadione-medium for 8 h enhanced the activity of MS. Thus, the glyoxylate shunt is crucial for providing the necessary intermediates for the NADH/NADPH conversion cycle.

## Discussion

The foregoing data point to a metabolic network dedicated to the conversion of NADH into NADPH in *P. fluorescens* as a consequence of oxidative stress. This is the first demonstration of a metabolic pathway that has as its main objective to generate NADPH with the concomitant reduction of NADH. NADPH is central to any anti-oxidative defense strategies in any organism as it is the ultimate power that fuels the reductive cellular processes. For instance, glutathione with the involvement of glutathione peroxidase mediates the neutralization of H_2_O_2_. However, without the regeneration of reduced glutathione, a process that necessitates the participation of NADPH, this anti-oxidative defence strategy will be ineffective. NADPH replenishes the reductive force of all enzymes that contribute to the diminution of intracellular oxidative tension [19].

In this instance, enzymes involved in disparate metabolic pathways partnered to create a novel metabolic network aimed at converting NADH into NADPH. PEPCK and PC are important components of gluconeogenesis. The latter provides oxaloacetate while the former helps generate phosphoenolpyruvate (PEP) that is eventually transformed into glucose [20]. In the menadione-challenged cells, these enzymes were uncoupled. While PC was upregulated, PEPCK was downregulated. However, the activity and expression of the glycolytic enzyme PK was enhanced. This allowed for any PEP formed to be utilized in ATP production with the concomitant generation of pyruvate, a substrate for PC. The latter participates in the carboxylation of pyruvate into oxaloacetate [21]. This ketoacid served as a sink for NADH. Indeed, MDH reduces oxaloacetate to malate producing NAD in the process. The pool of malate was further augmented by the upregulation of ICL and MS, two key enzymes of the glyoxylate cycle. This dicarboxylic acid is processed by ME to produce NADPH and pyruvate.

Hence, the channeling of pyruvate towards oxaloacetate production and the reduction of this ketoacid with the participation of NADH, provides an effective metabolic module to generate NADPH during oxidative stress. The plasticity of this network is evident as it borrows components from diverse metabolic pathways with the goal of increasing the pool of NADPH in an organism confronted with oxidative challenge. Furthermore, metabolic networks, like gluconeogenesis and the TCA cycle that are not critical for survival during this situation, are in essence markedly impeded. The changing flow of metabolites mediated by the downregulation and upregulation of some enzymes dedicated to the classical metabolic pathways helped the organism create a novel metabolic module designed to fulfill a specific objective, i.e., the transformation of NADH into NADPH. This is the first study demonstrating the involvement of a metabolic adaptation that resulted in diminishing NADH, a pro-oxidant coupled to an increase in NADPH, an anti-oxidant during oxidative stress. It is important to note that excessive NADH may help release Fe (II) from ferritin, increase the activity of respiratory complexes or such enzymes as xanthine oxidase with the further accentuation of oxidative stress [22]. Hence, the increased production of NADPH without the concomitant reduction in NADH formation may not be very effective. This metabolic network appears to attain both of these objectives.

Although there are numerous enzymatic systems that allow living organisms to generate this reductive moiety, these processes do not interact with each other and are usually confined to a specific environment in the cell. G6PDH is usually localized in the cytoplasm and is part of the pentose phosphate pathway [1]. This metabolic module can potentially generate two NADPH. However, ribose-5-phosphate or xylulose-5-phosphate is an important by-product that needs to be further transformed. If the cell is in a proliferative mode, ribose-5-phosphate can help generate deoxyriboses, moieties essential for the DNA replication [23]. On the other hand, under oxidative stress when the reduction in the concentration of ROS is a primary concern, the pentose has to be transformed into precursors that can be utilized in the further production of NADPH [24]. Hence, this metabolic network may not be fully self-sustaining; therefore, ineffective in combating oxidative stress. Even though G6PDH has been found to be uncoupled from the enzymes of the pentose phosphate pathway during oxidative challenge, the efficacy of this system depends on a constant supply of glucose-6-phosphate, an energy-rich compound. Furthermore, the product of this reaction 6-phosphogluconate needs to be further processed if the production of NADPH via G6PDH is to be effective. Hence, this metabolic pathway may not be entirely appropriate when the cell has to contend with an increasingly oxidative milieu.

The one-step enzymatic production of NADPH usually requires a constant supply of the substrates. ME, GDH and ICDH clearly belong to this category of NADPH producers. Pyruvate and α-ketoglutarate can be utilized for a variety of functions, including as ROS scavengers [6], [25]. We have recently shown how α-ketoglutarate can contribute to the homeostasis of ROS. Its modulation mediated by the TCA cycle may serve the dual purposes of nullifying ROS and mitigating the production of NADH, a pro-oxidant. This keto acid, as a consequence of its ROS-scavenging function, produces succinate, a metabolite that appears to have a signaling role [26]. Hence, this raises the possibility this one-step NADPH generator where succinate has an added role of promoting anaerobiosis may be very effective as an oxidative defence mechanism. However, it is important to point that the metabolic network uncovered in this study may provide the flexibility necessary for a cell to adapt whenever it is confronted with the challenge of an oxidative environment. This metabolic engine can be easily switched off/on by the upregulation and downregulation of the participating enzymes without major remodeling of its metabolic pathways as the metabolites involved can also fuel the “housekeeping” metabolic networks that are operative during normal conditions. Furthermore, this metabolic pathway has the added advantage of producing NADPH at the expense of NADH, a pro-oxidant, compared to the single-step NADPH generators.

**Figure 6:**
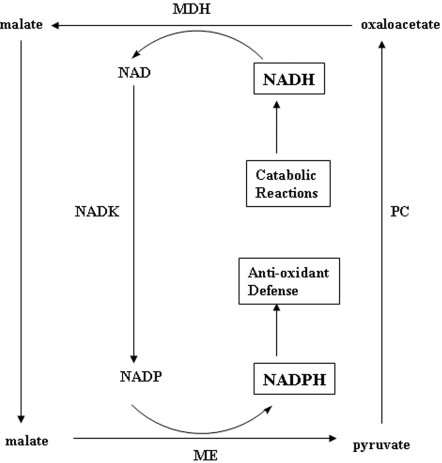
A novel anti-oxidantive defense strategy aimed at converting NADH, a pro-oxidant, into NADPH, an anti-oxidant. MDH = malate dehydrogenase, ME = malic enzyme, PC = pyruvate carboxylase, and NADK = NAD kinase.

The convergence of numerous enzymes from disparate metabolic pathways in an effort to create a metabolic network dedicated to fend a given cellular challenge may be at the core of all molecular adaptative and evolutionary processes. In this instance, the newly-found synergy, amongst the seemingly unrelated metabolic enzymes, ensures the survival of *P. fluorescens* assaulted by oxidative stress. This novel metabolic network completely redesigned the global metabolism of the organism. It prioritized the conversion of NADH to NADPH with the concomitant attenuation of gluconeogenesis and the TCA cycle, two metabolic pathways deemed onerous during oxidative stress. Hence, metabolic pathways have to be considered as very fluid and malleable systems whose discrete entities can partner with each other to create unique metabolic modules aimed at providing a certain degree of evolutionary flexibility to all life forms. These partnerships may be modulated by fluxes in the internal and external environment. In this instance, *P. fluorescens* appears to have utilized this strategy to survive an oxidative environment by creating a unique metabolic network tailored to transform a pro-oxidant, NADH into NADPH, an anti-oxidant (Figure 6).
