# Correction: A Novel Strategy Involved in Anti-Oxidative Defense: The Conversion of NADH into NADPH by a Metabolic Network

**DOI:** 10.1371/annotation/5fac086b-3806-4aa9-a5c5-2611b3355f8f

**Published:** 2008-07-29

**Authors:** Ranji Singh, Joseph Lemire, Ryan J. Mailloux, Vasu D. Appanna

The word "in" was omitted from the article title, and the title appears incorrectly in the citation as well. The correct title is: "A Novel Strategy Involved in Anti-Oxidative Defense: The Conversion of NADH into NADPH by a Metabolic Network"

